# 360° Map Establishment and Real-Time Simultaneous Localization and Mapping Based on Equirectangular Projection for Autonomous Driving Vehicles

**DOI:** 10.3390/s23125560

**Published:** 2023-06-14

**Authors:** Bo-Hong Lin, Vinay M. Shivanna, Jiun-Shiung Chen, Jiun-In Guo

**Affiliations:** 1 Institute of Electronics, National Yang Ming Chiao Tung University, Hsinchu 30010, Taiwanjiguoccu@gmail.com (J.-I.G.); 2 Lite-On Technology Corporation, Taipei 11492, Taiwan; 3 Pervasive Artificial Intelligence Research (PAIR) Labs, National Yang Ming Chiao Tung University, Hsinchu 30010, Taiwan

**Keywords:** equirectangular projection, panorama, road object detection, 360°

## Abstract

This paper proposes the design of a 360° map establishment and real-time simultaneous localization and mapping (SLAM) algorithm based on equirectangular projection. All equirectangular projection images with an aspect ratio of 2:1 are supported for input image types of the proposed system, allowing an unlimited number and arrangement of cameras. Firstly, the proposed system uses dual back-to-back fisheye cameras to capture 360° images, followed by the adoption of the perspective transformation with any yaw degree given to shrink the feature extraction area in order to reduce the computational time, as well as retain the 360° field of view. Secondly, the oriented fast and rotated brief (ORB) feature points extracted from perspective images with a GPU acceleration are used for tracking, mapping, and camera pose estimation in the system. The 360° binary map supports the functions of saving, loading, and online updating to enhance the flexibility, convenience, and stability of the 360° system. The proposed system is also implemented on an nVidia Jetson TX2 embedded platform with 1% accumulated RMS error of 250 m. The average performance of the proposed system achieves 20 frames per second (FPS) in the case with a single-fisheye camera of resolution 1024 × 768, and the system performs panoramic stitching and blending under 1416 × 708 resolution from a dual-fisheye camera at the same time.

## 1. Introduction

In indoor, aerial, or underwater vehicles, simultaneous localization and mapping (SLAM) [1,2,3,4] is the first problem encountered when unmanned aerial vehicles (UAVs) [5] or robots [6] enter an unknown environment. SLAM is a concept for constructing unknown environment models and estimating the sensor motion during the movements. The basic SLAM system includes sensing, mapping, localization, and optimization. This concept was firstly proposed for achieving autonomous control of robots. Over the past few years, vision cameras have been widely used as SLAM sensors because of their low cost and simple configuration.

Visual SLAM (V-SLAM) [7,8,9] tries to recognize a scene using images employing various computer-vision algorithms with motion, corner, edge, and other features. The challenge of V-SLAM is that the field of view (FOV) of general monocular cameras is limited [10,11], covering barely 50° to 70°. If there are significant changes in the scene, particularly in the continuous frames when the camera or the object in the scene moves, the position or posture of the object in previous frame may disappear in the current frame.

To overcome such difficulties, an equirectangular panorama, which has the benefit of the wide 360° FOV and the popular projection method, are adopted as the input image type of the proposed vision SLAM (V-SLAM) system. However, since the feature-based SLAM [7,12,13] is the chosen algorithm in this paper for high luminance noise immunity, the image size of the panorama is huge, and the feature extraction and feature matching may require a higher processing time. Therefore, the perspective transformation with a given yaw degree is adopted. These features combined together form the proposed 360° real-time vision SLAM system based on the equirectangular projection.

## 2. Related Work

This section discusses some of the state-of-the-art SLAM methods.

### 2.1. Existing SLAM Designs

**Figure 1:**
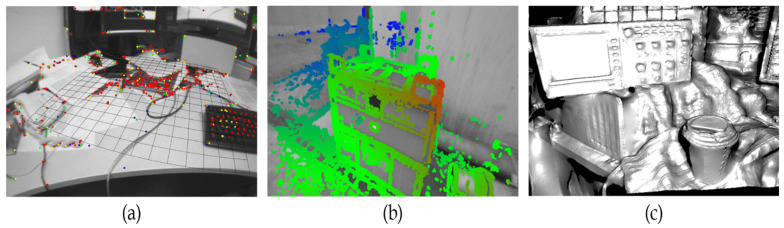
(**a**) The sparse map generated by PTAM [8]. (**b**) The semi-dense map created by the LSD-SLAM [9]. (**c**) The dense map in the DTAM [10].

According to the size of the area used in a perspective image, the maps of SLAM systems are divided into two major categories, namely, sparse and dense maps. The sparse methods use only a small, selected subset of the pixels in each received frame, whereas the dense methods use most of the pixels present in an image. Due to a different number of pixels in an image used by sparse and dense methods, respectively, the properties of maps generated from the two methods are different. The maps built from the dense methods provide more details of the scene than those in the sparse maps, and high computational hardware is usually required to meet real-time processing demands. On the other hand, the type of sparse maps is points clouds, which is mainly used to track the camera pose, i.e., localization and a rough representation of the scene. Figure 1 depicts the difference between maps built from sparse and dense methods. Figure 1a shows to the results of sparse maps with selected subset pixels highlighted in red, Figure 1b is result of the semi-dense maps highlighting the selected subset of pixels in green and blue and Figure 1c is the result of the dense maps in which most of the pixels in the frame are selected as shown.

**Figure 2:**
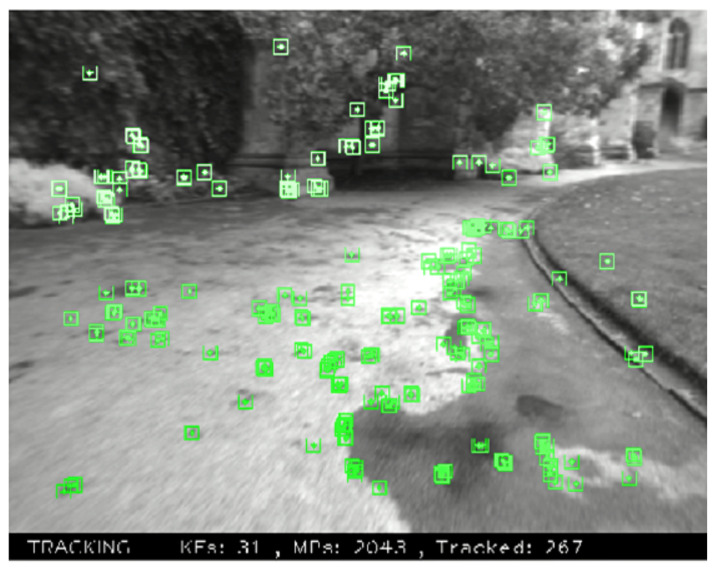
ORB feature points in the ORB-SLAM [15] system.

The existing SLAM system design can be broadly classified into two types: (i) feature-based methods, and (ii) direct methods. The feature-based methods extract feature points first, using these feature points to build the map and estimate the camera pose. These feature points can be common geometric features such as corner points, or more complicated features such as SIFT [10], SURF [11], ORB [12], FAST [13], ORB [14] or ORB-SLAM [15], as shown in Figure 2. Feature-based methods provide a higher tolerance range in brightness, as the intensity values of the pixels are not directly used. Despite this, the feature extraction processes are highly time-consuming.

In contrast, the direct methods use the intensity values of the pixel directly instead of extracting the features, and it is implied that direct methods leave more time for other computing processes and maintain the same performance as that of the feature-based methods. The direct methods optimize the map and the camera pose together, and attempt to reconstruct the environment. However, the main disadvantage of the direct method is the higher sensitivity to ambient light.

#### 2.1.1. Feature-Based Methods

Feature-based methods can be broadly classified as two types: filter-based and bundle adjustment (BA)-based [16] methods.

Among the filter-based V-SLAM algorithms, MonoSLAM [17,18] is considered the classical method. By using an extended Kalman filter (EKF), an unknown 3D environment model and camera motion are simultaneously estimated, and state vectors are used to represent the 3D positions of the feature points. In a prediction model, a constant velocity motion is assumed, and feature point tracking results are used as an observation. The map is initialized by observing a known object where a global coordinate system is defined. In summary, MonoSLAM is composed of (i) a known object to carry out the map initialization, followed by (ii) using EKF to estimate the 3D positions of the feature points and camera pose. The disadvantage of MonoSLAM is that there is a positive correlation between the computational cost and the region of an environment. It is hard to achieve real-time computation in large environments, because of the size of a state vector and the large number of feature points.

In order to reduce the computational complexity of MonoSLAM, the parallel tracking and mapping (PTAM) [8] method separates the mapping and the tracking into different threads. Since these two threads are executed in parallel, the computational cost of the tracking and mapping do not affect each other. Therefore, the bundle adjustment, which is of high computational complexity in the optimization, is used in mapping the estimates to accurate 3D positions of the feature points, while the tracking estimates the camera pose in real-time. PTAM is the first SLAM algorithm that incorporates BA into real-time V-SLAM systems. Since the proposal of PTAM, most V-SLAM algorithms have followed the multi-threading concept.

The five-point algorithm [19] is adopted in PTAM to perform the map initialization. In the tracking, the camera poses are estimated from 2D–3D correspondences by projecting the mapped points onto an image and using the texture matching. In the mapping, 3D positions of new feature points are estimated by triangulation and optimized by local/global bundle adjustment with keyframes. When a large disparity between the input frame and the one of the keyframes is measured, a new keyframe created by the current input frame is inserted into the map. However, the new version of PTAM introduces a re-localization algorithm [20] in the tracking process. A randomized tree-based feature classifier is used for searching the nearest keyframe of an input frame. To sum up, the PTAM is composed of the four components listed below.

(i) The five-point algorithm [19] is adopted to do the map initialization.
(ii) Camera poses are estimated from corresponding feature points between the input image features and map points.
(iii) 3D positions of feature points are estimated by triangulation and optimized by BA.
(iv) A randomized tree-based searching [20] is adopted to recover the tracking process.

Compared to MonoSLAM, the PTAM system is able to handle thousands of features by separating the mapping and the tracking into different parallel threads.

#### 2.1.2. Direct Methods

In direct tracking and mapping (DTAM), the mapping is performed using multi-baseline stereo [21], and the map is optimized by considering space continuity [22] so that 3D positions of all the pixels can be estimated. The tracking is performed by comparing the input image with synthetic images created from the reconstructed map. In other words, an image is registered in 3D map of the environment and the registration process is efficiently implemented on GPU in DTAM. The initial depth map is calculated by using a stereo measurement like in PTAM. To summarize, the DTAM is composed of the following components:

(i) Stereo measurement is adopted to perform the map initialization.
(ii) Camera motion is estimated by synthetic images generated from the reconstructed map.
(iii) The depth of every pixel is estimated using multi-baseline stereo, and optimized by considering space continuity.

From the above discussions of the sparse and dense types of SLAM maps and the existing SLAM algorithm designs, it is noted that the feature-based methods are good for applications that require a high luminance noise immunity.

In addition to the aforementioned state-of-the-art methods, SLAM has been employed in developing autonomous indoor robots [23,24] that can be used for numerous applications ranging from exploring and examining the closed space to unpacking and ordering of goods to cleaning of the premises. The visual SLAM method can be used to build navigation aids for blind people [25], which is similar to the operation of Google maps using a pre-established environmental maps and landmarks. Additionally, the SLAM can be implemented using dynamic field theory (DFT) [26] but it demands large memory and its associated computation overhead.

Some of the latest publications of SLAM methods are presented herein. Caracciolo et al. [27] presented open visual simultaneous localization and mapping (OpenVSLAM), which is derivative of ORB-SLAM. In this method, the occupancy grid map (OGM) is generated using the keyframes and landmarks obtained using SLAM maps followed by using a path algorithm employing the informed search algorithm which can function only in the known environments. On the other hand, Zhao et al. [28] proposed a fisheye image calibration method based on a 3D calibration field, which is evaluated using ORB-SLAM3. Summarizing the applications of the SLAM-based methods, Roli et al. [29] discussed different probable uses of SLAM-based devices in different fields including autonomous vehicles, healthcare, security and surveillance, and industrial automation. The other state-of-the-art methods that direct in the evaluation of the SLAM based methods are presented in [30,31].

The contributions of the proposed method of this paper are as follows:

(i) The proposed method adopts the perspective transformation with any yaw degree which overcomes the drawback in the previous methods when dealing with huge panoramic images.
(ii) Since the proposed method adopts the perspective transformation with any yaw degree, the feature extraction and feature matching are faster compared to the state-of-the-art methods. However, in the process of localization the proposed SLAM algorithm, the perspective transformation is performed only on a specific region of panorama. The other regions which may also possess more, meaningful details that can be used for camera pose estimation is not covered in the proposed algorithm.

The next sections describe the procedures and methods, as well as the corresponding results of the proposed method. 

## 3. Proposed Model

**Figure 3:**
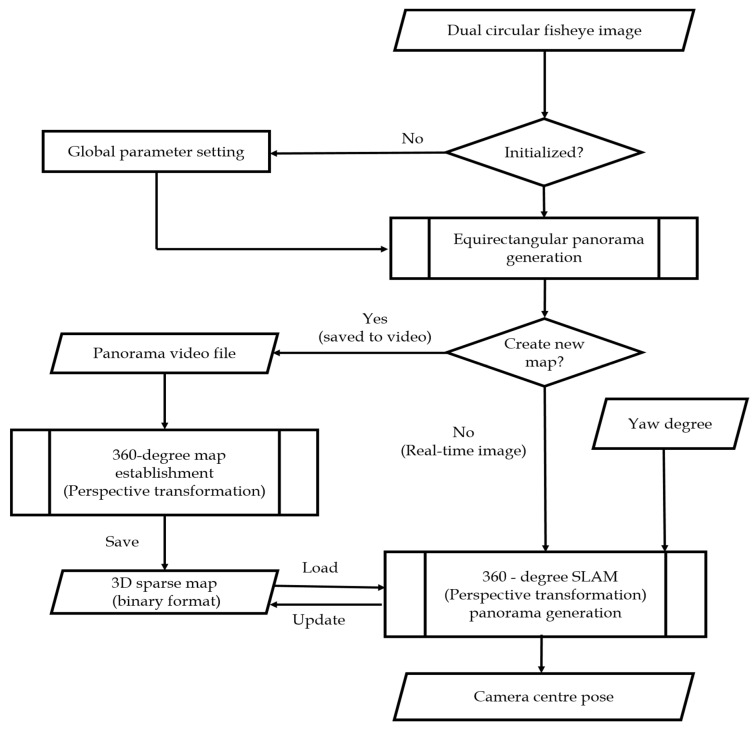
Flowchart of the proposed algorithm.

Figure 3 presents the flowchart of the proposed algorithm comprising the 360-SLAM mode and map establishment mode. In the 360-SLAM mode, the system is initialized with the global parameter setting as detailed in Section 3.1, followed by the equirectangular panorama generation discussed in Section 3.2. Then, a new map is created with a perspective transformation panorama generation toward the camera central pose as outlined in Section 3.3. On the other hand, in the map establishment mode, the new map is created due to a panorama video file with the establishment of 360° perspective map establishment saving the 3D sparse map in the binary format, as explained in Section 3.4. The overall processes of the proposed method consist of three basic parts. The first part is the generation of an equirectangular panorama comprising circular fisheye calibration, stitching, and blending, during which, if a look-up table (LUT) is not built, the process flow loads the global parameters to build the LUT. The second part is the establishment of a 360° map, which uses the stitched result to build and save the 3D sparse map. The third and the last part is the generation of a 360° SLAM that outputs the camera center pose and updates the map in real time with a preloaded 360° map. The system initialization, which includes global parameter setting, makes the proposed system suitable for various scenes.

Equirectangular projection is adopted to display the panoramic stitching result and is retained as the image input type of the 360° SLAM because of its universality. There are two operating modes in proposed system. For the map-building mode in which the multi-camera device captures the image, only the equirectangular panorama video file is generated first since map building takes more time. Then, the 360° map is built from video scenes and saved in a binary format in order to reduce the map size and the time taken to load the map. For the SLAM mode, the panoramic image is directly used for localization.

The next sections discuss the global parameter setting, the circular fisheye calibration, dual-fisheye panorama stitching, the proposed 360° map establishment method, and the overall proposed 360° SLAM algorithm.

### 3.1. Global Parameter Setting

**Figure 4:**
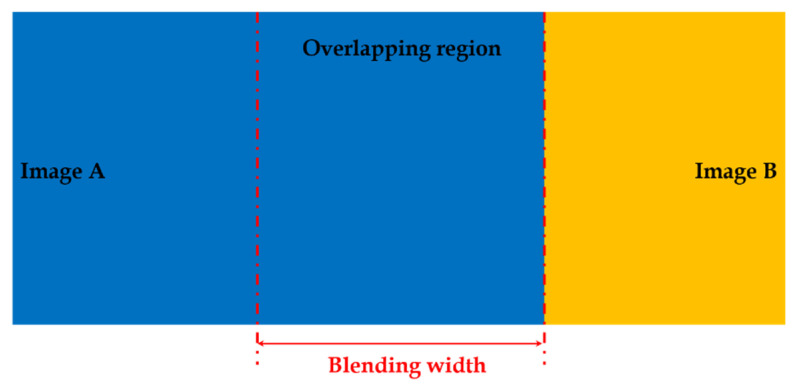
Blending width illustration.

Parameter initialization makes the system suitable for various scenes and closed to user interface. There are three types of parameter settings. Initially, a map name is set with the filename extension ‘bin’. If the map name already exists, the system loads the map whereas, if the system cannot find the corresponding map name, it creates a new map under a new filename. In the proposed algorithm, perspective transformation is applied and related to five parameters: (i) horizontal field of view, (ii) yaw angle, (iii) pitch angle in degree unit, (iv) output width, and (v) output height in pixel unit. Then, a respective basic camera input image’s height and width are set. In dual-fisheye stitching cases, the calibration parameters are set for both the front and the rear circular fisheyes such as field of view in degrees, center coordinates, and radius of the circle in pixels. A blending process is adopted to reduce the seam of the overlapping region between different images so that the blending width is required to set an appropriate value, as shown in Figure 4. If the blending width is too wide, the computation complexity of blending will be higher, and blending region will be beyond the overlapping area, whereas, if the blending width is narrow, the seam will be more obvious.

**Figure 5:**
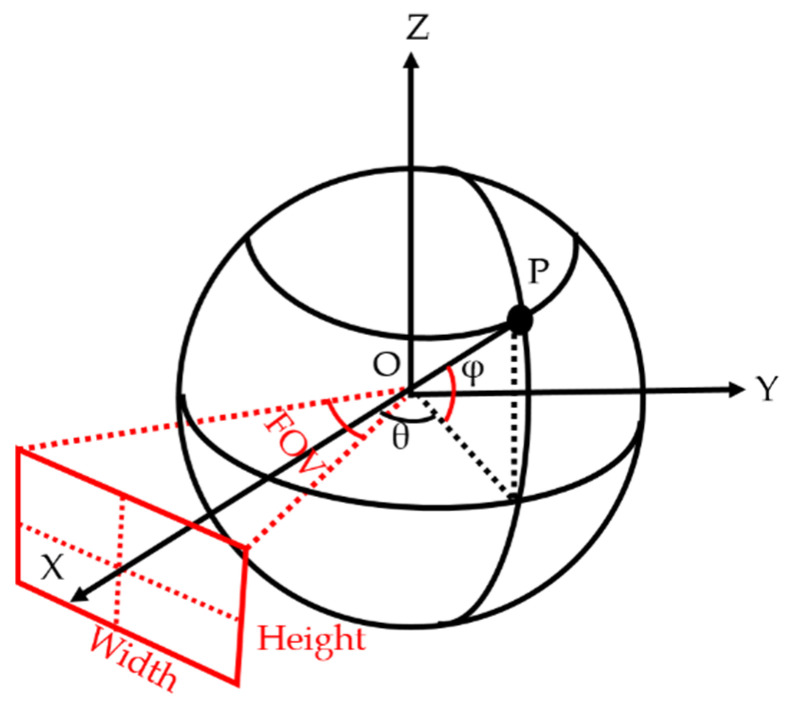
Illustration of perspective transformation parameters.

As shown in Figure 5, the red rectangular area is decided by the “width” and “height” parameters is a perspective transformation output image plane, with the normal vector always passing through the spherical and image plane center point. With a fixed value of width and height, a smaller value of FOV allows obtaining more details from the output image. In Figure 5, point *P* is the center of the image plane, *φ* is the pitch angle, and *θ* is the yaw angle.

### 3.2. Equirectangular Panorama Generation

**Figure 6:**
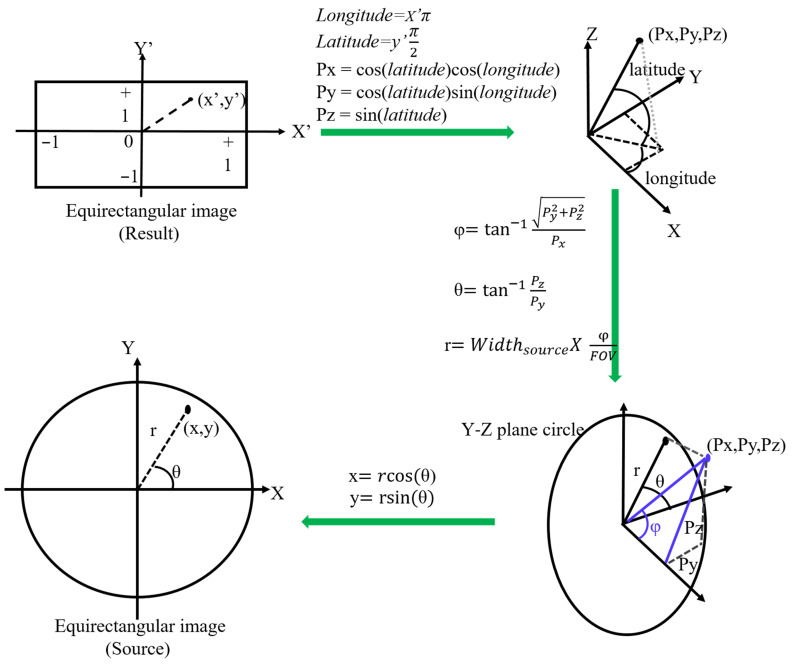
Equirectangular projection diagram.

Circular fisheye lenses are designed to cover the hemispherical view in front of the camera, and stitching two hemispherical images is important for panoramic images. The first step of stitching is unwarping the circular images, and the look-up table is generated according to the formulas illustrated in Figure 6. The final image projection type is equirectangular, and each pixel must be assigned a value from the pixel inside the circular image. Therefore, the look-up table needs to be inversely calculated in sequence following the green arrows in Figure 6.

**Figure 7:**
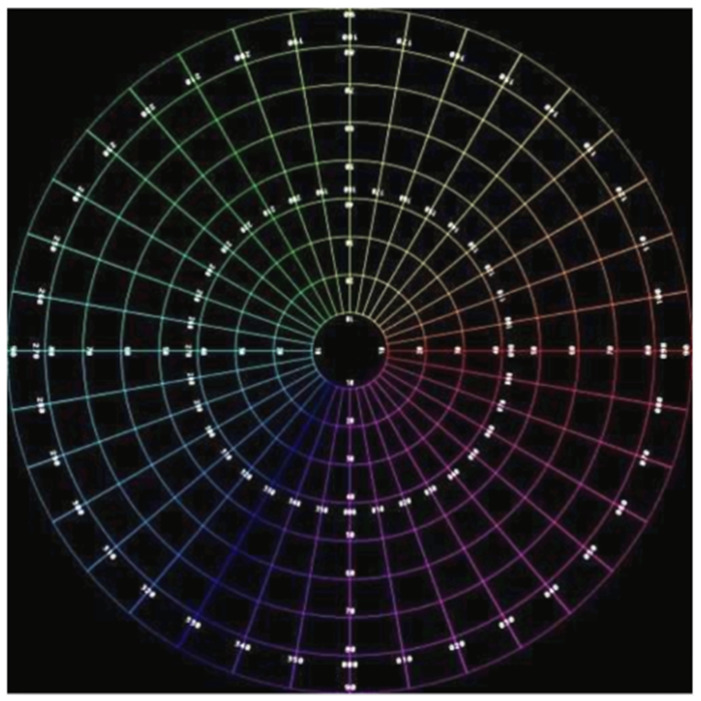
A 180° circular fisheye image with degree marker.

**Figure 8:**
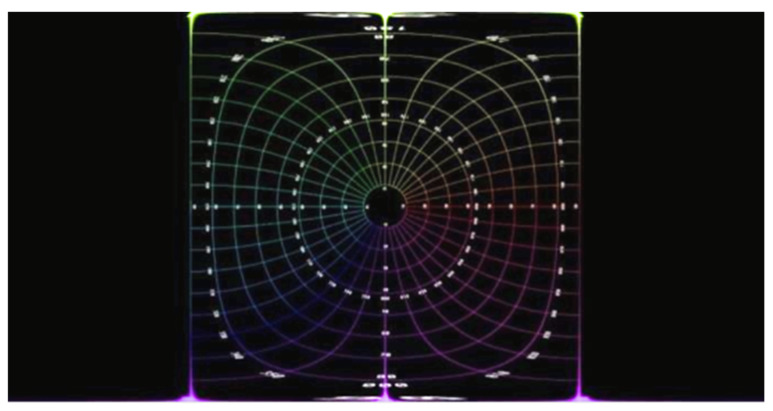
Equirectangular projection image of 180° circular fisheye.

Figure 7 depicts an ideal equidistance circular fisheye image with 180° FOV. For the verification of the proposed unwarping algorithm, a raw image is marked with a value every 10°. Figure 8 is an unwrapped result, and the 90° circle in the raw image is projected to a square in the output image. Due to the property of the equirectangular projection, extreme distortion occurs at both the top and the bottom regions. As shown in Figure 8, the four corners of the square are not perfect but still accepted, as the top and bottom regions of the unwrapped image are seldom considered prominent.

**Figure 9:**
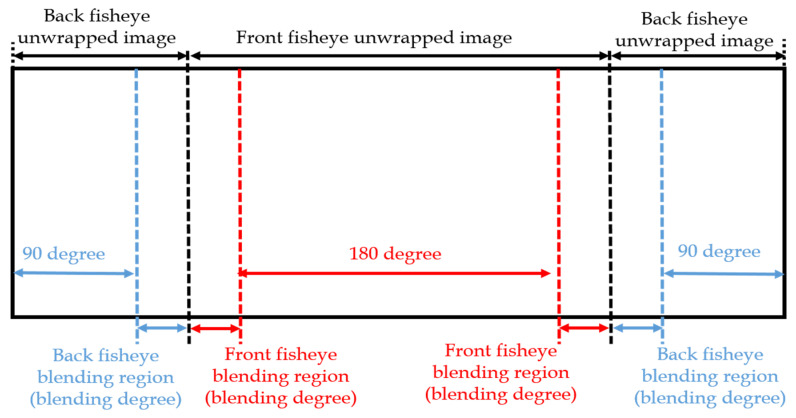
The proposed method of the look-up table.

Generating two unwrapped images and stitching them is the general method. However, many ineffective pixels are located at both the sides in the unwrapped image; thus, most values of the unwrapped LUT are meaningless. In order to speed up the remapping process, the proposed algorithm combines two unwrapped images into one image by using only one LUT as shown in Figure 9, and the result is presented in Figure 12.

**Figure 10:**
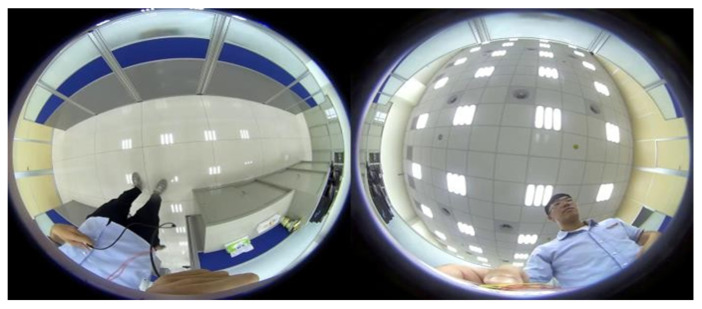
Dual back-to-back fisheye raw images.

**Figure 11:**
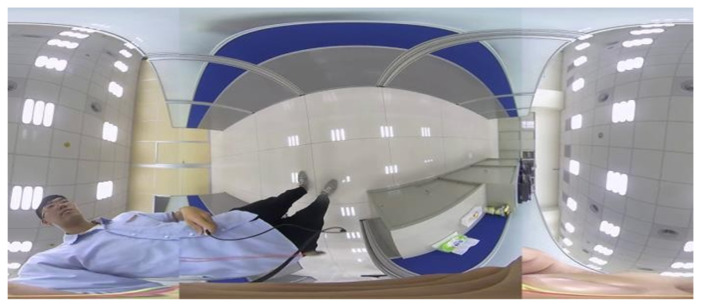
Equirectangular panorama without blending.

**Figure 12:**
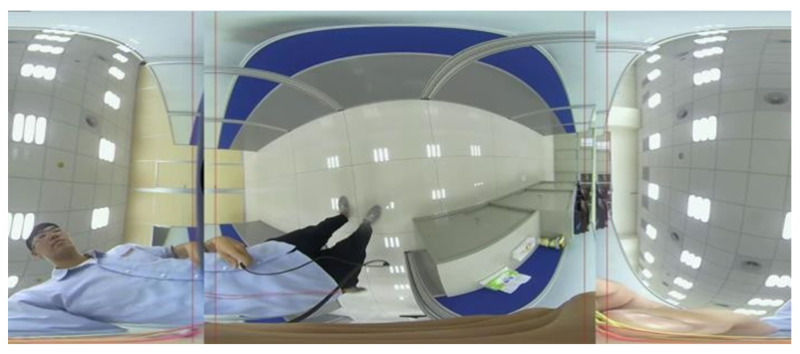
Semi-stitching image.

**Figure 13:**
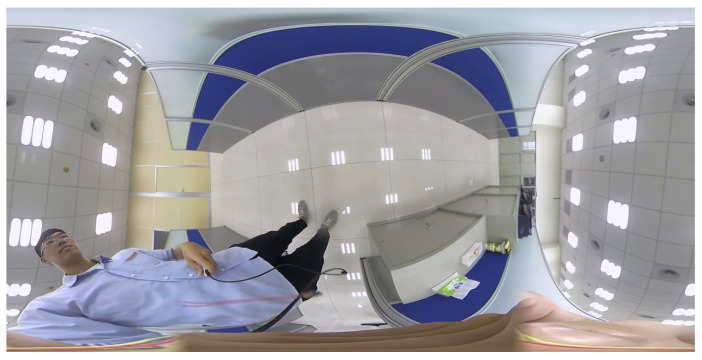
Equirectangular panorama with blending.

Figure 10 depicts the dual back-to-back fisheye raw images and the direct equirectangular panorama unwrapping from Figure 10 are as in Figure 11. Without blending, the seam is obviously visible. Figure 12 presents the semi-stitching result generated by the proposed method, for preparing the input images of blending. The final equirectangular panorama with blending is shown in Figure 13.

### 3.3. Perspective Transformation

**Figure 14:**
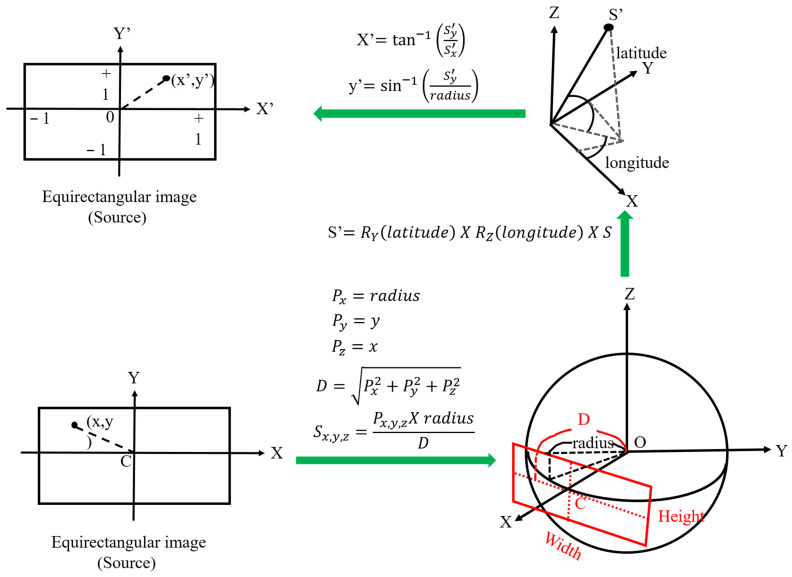
Perspective transformation diagram.

Equirectangular projection is a popular projection method whose output image format is supported by many panoramic cameras or video editors. It has a fixed width-to-height ratio of 2:1 despite which the top and bottom areas occupy most of the display region resulting in high distortion. The image size of the thus obtained panorama is huge; hence, the time consumed in feature extraction from such images is usually considered longer for real-time processing. Perspective transformation is adopted to convert panoramas to planar images with any yaw degree given by the proposed system. Figure 14 illustrates the procedure of perspective transformation, and this conversion is used in both map establishment and localization in the proposed system.

**Figure 15:**
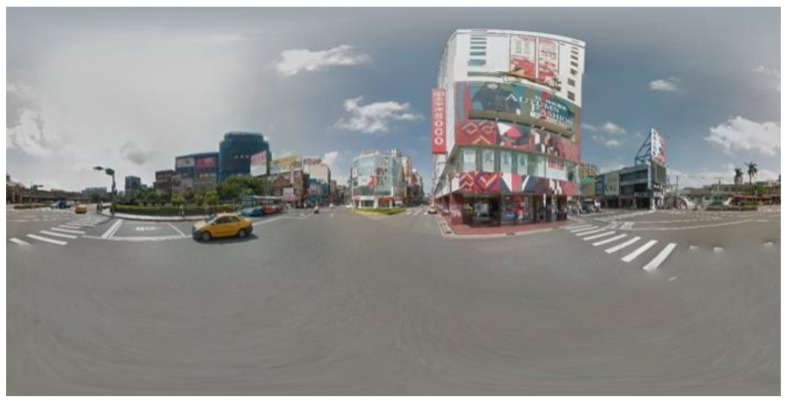
Equirectangular panorama source.

**Figure 16:**
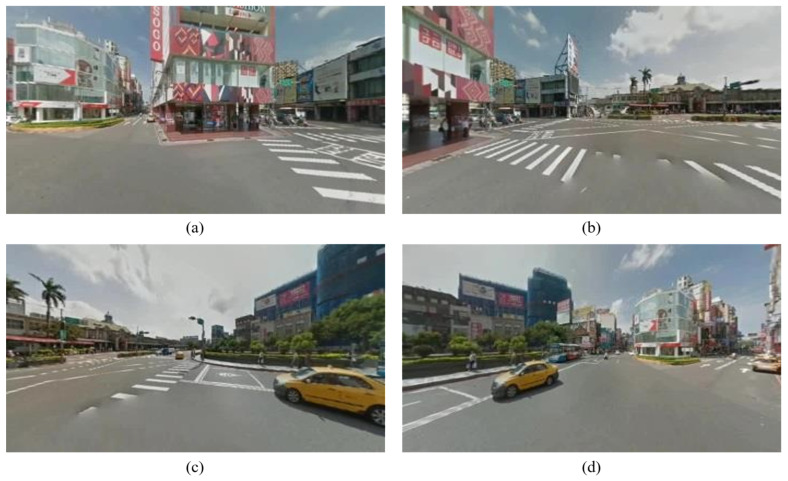
Output images of perspective transformation with yaw degree given: (**a**) 45°; (**b**) 135°; (**c**) 225°; (**d**) 315°.

Figure 15 presents the source panorama image, and the result pictures of perspective transformation with yaw degree given are shown in Figure 16.

### 3.4. 360° Map Establishment and Automatic Yaw Degree Rotation

Since a scene to be built is viewed only once in the proposed method and the single perspective image is unable to cover the 360° view, the perspective images of each respective yaw degree are required to be input in the proposed system for constructing the 360° map. However, manually rotating the yaw degrees in the whole process is time-consuming; thus, the proposed system supports the automatic yaw degree rotation by user-defined parameters.

In the process of 360° map establishment, the panoramic video needs to be played more than once. In order to maintain the frame continuity, the panoramic video is played forward and backward since the scenes at the start and end of a video are different. In other words, if the panorama video is always played forward repeatedly, the camera pose may get lost when the video ends. At the same time, the proposed system performs the re-localization, and the camera pose obtained from the re-localization may not be as accurate as the pose obtained from the tracking.

**Figure 17:**
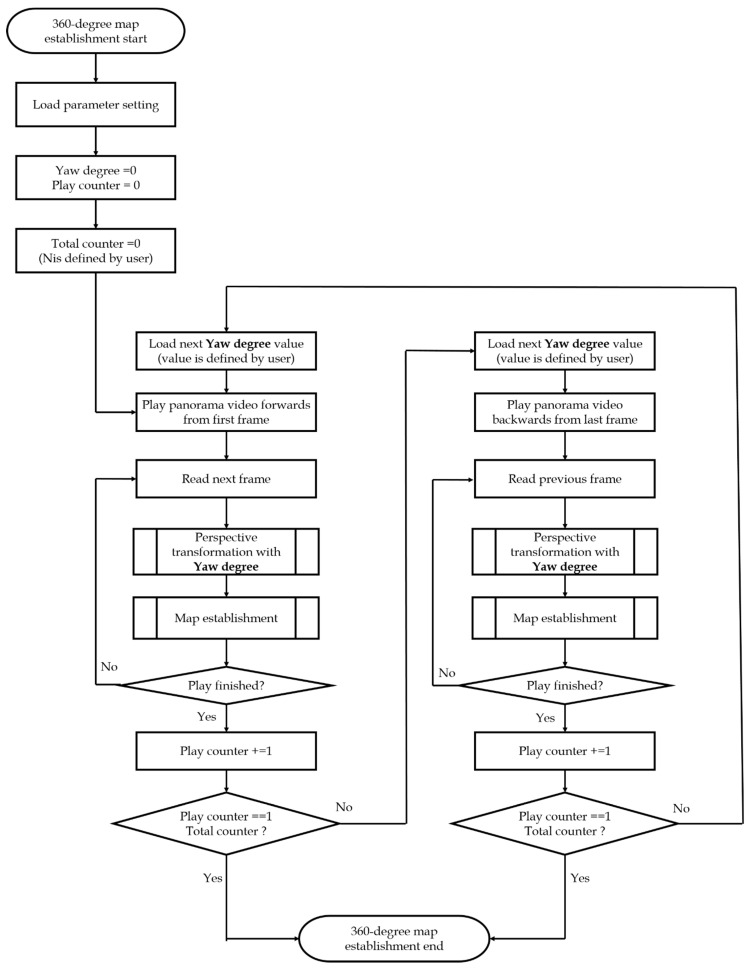
Proposed 360° map establishment flow.

Figure 17 presents the steps followed in updating the yaw degree. Before playing the video every time, the yaw degree is loaded and updated for generating the corresponding perspective image. In addition, compared to the monocular cameras, the act of going through the scene just once indicates that the time required for recording a scene can be reduced. When the video is played in the normal forward mode from the first frame, the yaw degree is used while reading each frame to do the perspective transformation with the yaw degree resulting in the map establishment. Similarly, when the video is played in the reverse mode from the last frame, the same yaw degree is used to do the perspective transformation, with the yaw degree resulting in the map establishment as depicted in Figure 17.

**Figure 18:**
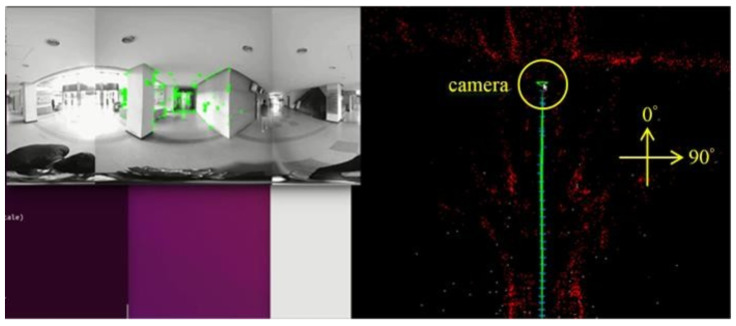
Map establishment at 0° yaw.

**Figure 19:**
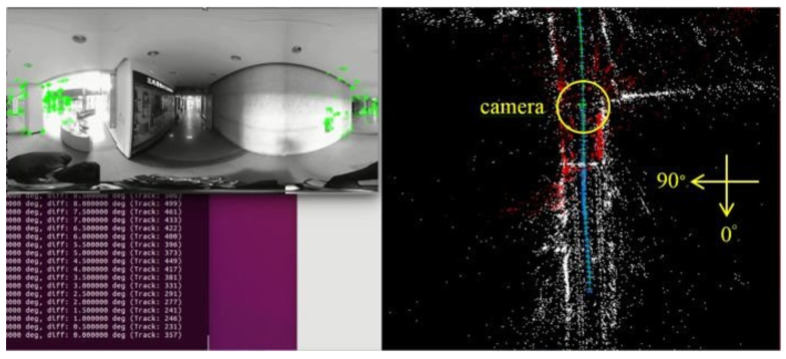
Map establishment at 180° yaw.

**Figure 20:**
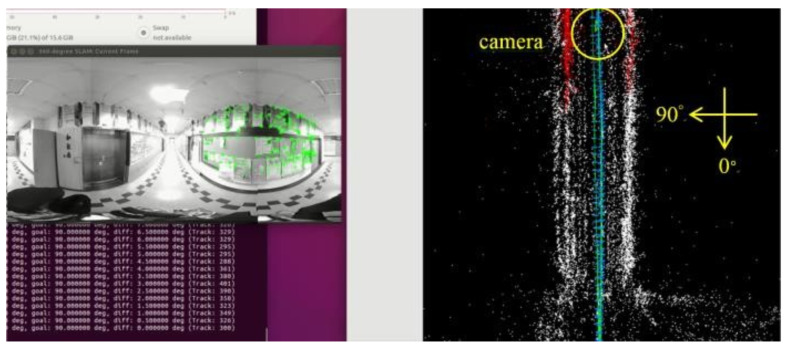
Map establishment at 90° yaw.

**Figure 21:**
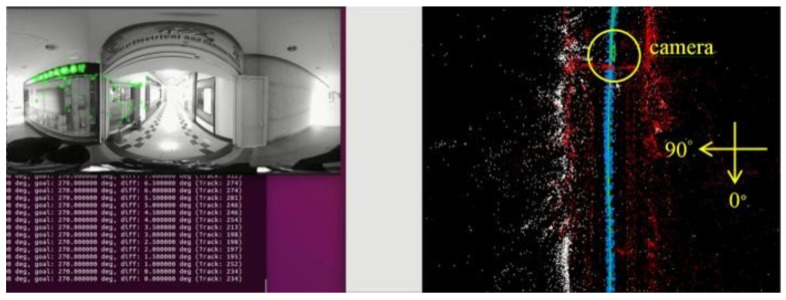
Map establishment at 270° yaw.

**Figure 22:**
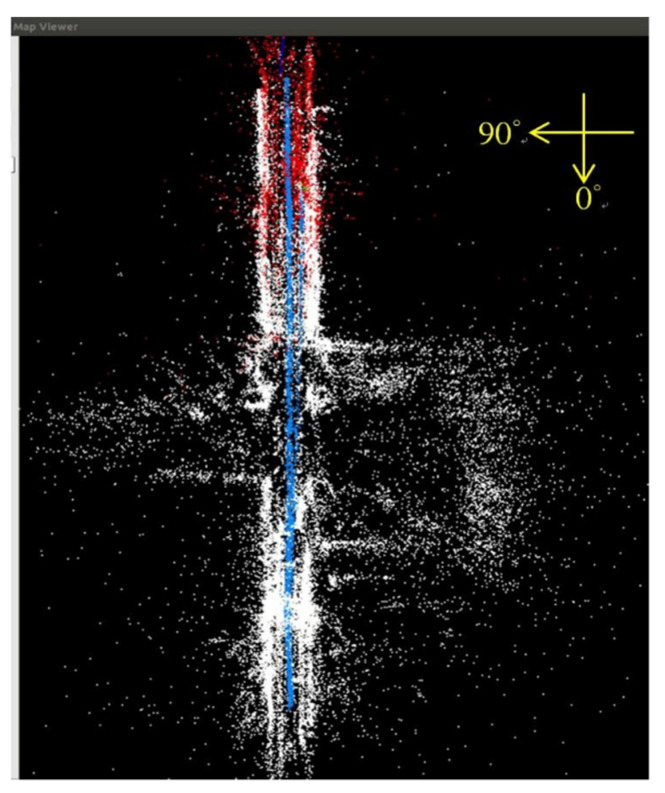
Result of 360° map establishment.

Figure 18, Figure 19, Figure 20 and Figure 21 show that, in the proposed method, the panorama video is alternatively played forward and backward for a total of eight times, and the yaw degree is sequentially set to 0°, 180°, 315°, 135°, 90°, 270°, 45°, and 225° with the aim of simulating the front view of the camera captures moving back and forth. Figure 22 depicts the result of the 360° map constructed by the proposed method.

### 3.5. Map Saving, Loading, and Online Updating Function

The drift error and the initialization are the two most important factors leading to the system requiring the loading map functions to be saved.

Drift error is a common problem in all SLAM systems. In SLAM algorithms, the camera pose of the current frame is estimated from the past frames, and there will be slight deviations in each frame. The errors in the past frames not only influence the measurement of the current frame but the errors are also accumulated, resulting in the drift errors. Without other sensors, such as inertial measurement unit (IMU), global positioning system (GPS), or light detection and ranging (LiDAR), the drift error is hard to correct. However, the SLAM system can reduce the drift error by comparing the similarity between the current frame and past keyframes. If the similarity exceeds a certain threshold chosen experimentally, it can be considered as detecting the loopback, also called loop closure in the SLAM system. If the system can load the map that has already finished performing the loop closure when the map was being built, the camera posing errors everywhere will be reduced, which results in increased accuracy of the localization.

Then, the system cannot guarantee that the place of finishing initialization is the same as in the real world each time. For autonomous driving vehicles, robots, or UAVs, it is difficult to determine the position, especially indoors, whereas the GPS can be used outdoors with accuracy levels on the order of 5–10 m. The centimeter-accuracy GPS can also be used but it is much more expensive than the cameras. If the map is loaded in the beginning, the proposed SLAM system can perform the re-localization and roughly yield the same camera pose every time.

For the above reasons, the map saving/loading functions need to be implemented. Furthermore, in order to reduce the time consumed in saving and loading map, the proposed system generates the 360° map that includes the important information only, such as keyframe databases and map points in the binary format.

**Figure 23:**
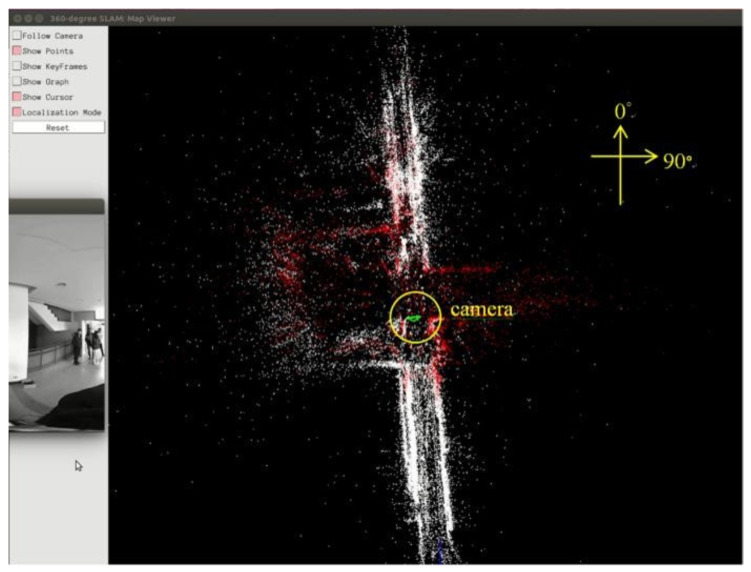
Screenshot of loading 360° map in the test video.

Figure 23 presents the 360° sparse map loaded in the test video scene. In Figure 23, the white points represent the total saved map points, red points are the referenced map points by the camera, and the green triangle is the camera.

In practice, the real scene may change every day, and the view of camera in localization mode will be slightly different from the scene of map building. In order to ensure that the map includes the latest scene features, online updating of the map is necessary. Sometimes, the scene may only temporarily change, and, in special cases, online updating is needed, but it will not be saved. Therefore, the proposed method has four operation modes, as presented in the Table 1.

**Figure 24:**
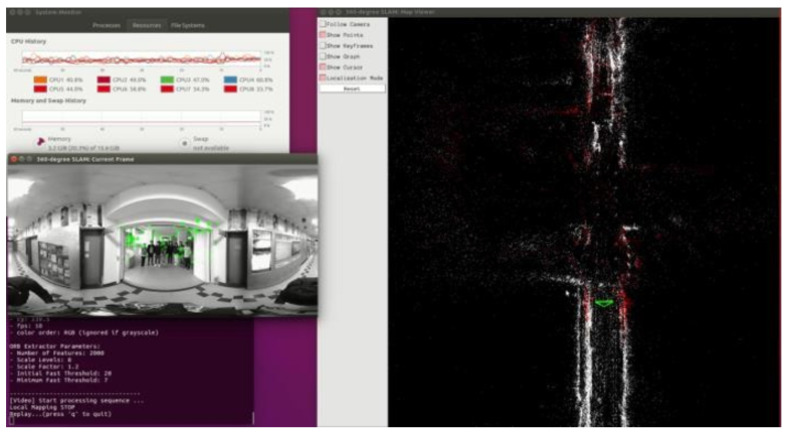
Screenshot of 360° SLAM system in localization mode.

As shown in Figure 24, the proposed system uses the equirectangular panorama as the input image displayed in the window of “360° SLAM: Current Frame”, and works in localization mode. The corresponding camera pose is a green triangle displayed in the window of “360° SLAM: Map Viewer.”

## 4. Implementation and Experimental Results

### 4.1. Implementation

**Figure 25:**
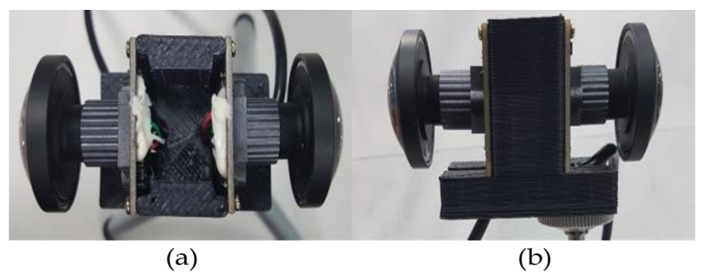
Dual back-to-back fisheye cameras.

In the implementation of the proposed method, the test videos were taken from the open datasets, and the hobby scenes were captured using dual back-to-back fisheye cameras (Figure 25). Figure 25a is top-view and Figure 25b is the side-view. The global parameters employed in the proposed system are presented in Table 2.

Table 3 lists the specifications of the desktop computer utilized to develop and verify the proposed method rapidly. Around 1600 frames of videos are collected using the dual-fisheye cameras for testing the proposed algorithm. Under a panoramic resolution of 1416 × 708 and without GPU acceleration, the proposed method achieved a performance of 20 fps.

The proposed algorithm was also implemented on the nVIDIA Jetson TX2 embedded system with specifications listed in Table 4 mounted on a wheelchair. The nVIDIA Jetson TX2 is a preferred embedded system for autonomous driving vehicles due to its low power consumption, controller area network (CAN) bus, 256 CUDA core GPU, multicore processor, and real-time processing efficiency. The wheelchair mounted with the nVIDIA Jetsion TX2 to implement the proposed method was moved at a speed of 3–4 km/h (i.e., ~0.8–1.1 m/s), and the angular resolution was 1° in building the 360° map for SLAM.

### 4.2. Evaluation

**Figure 26:**
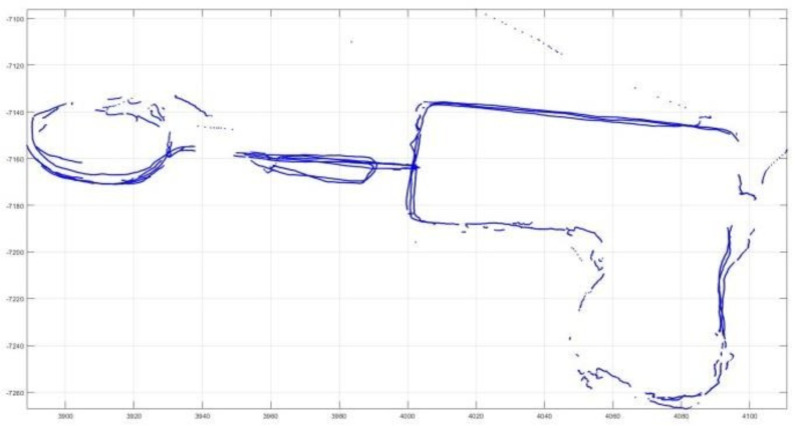
Fragmented GPS data provided by dataset.

The open dataset consisted of a few panoramic images with the ground truth. New College Datasets [15] was the open dataset used as the input, consisting of outdoor panoramic image sequences with the resolution 2048 × 1024. However, due to the fragmented GPS shown in Figure 26, achieving reliable ground truth was not feasible.

**Figure 27:**
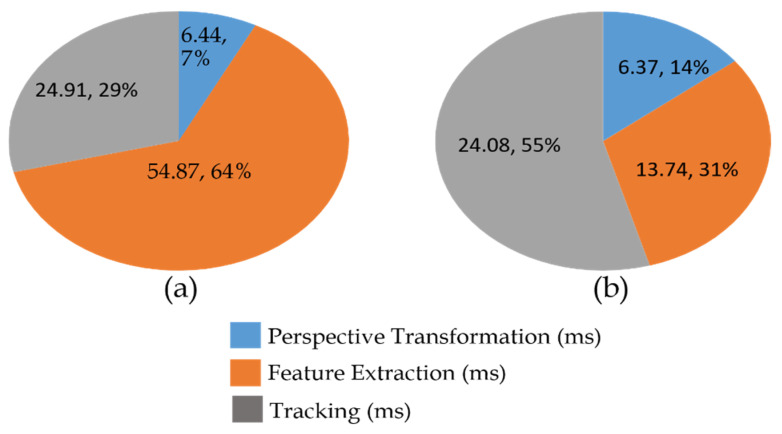
Timing measurement of the proposed method: (**a**) CPU version; (**b**) GPU version.

**Figure 28:**
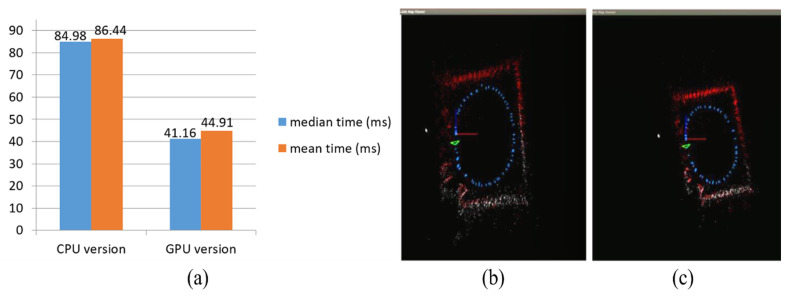
(**a**) Total time statistics of the proposed method in each frame. (**b**) Map result of the proposed method in CPU version. (**c**) Map result of the proposed method in GPU version.

The oriented fast and rotated brief (ORB) feature extraction of the proposed method was implemented with GPU acceleration, and the results are as shown in Figure 27. The time spent on the feature extraction was sped approximately fourfold, from an average of 54.87 ms to 13.74 ms. In addition, the total time statistics and the map result of the CPU and GPU versions are presented in Figure 28a–c. The median and mean time spent on each frame of CPU version was almost twofold longer than the GPU version.

#### 360° SLAM Ground Truth

**Figure 29:**
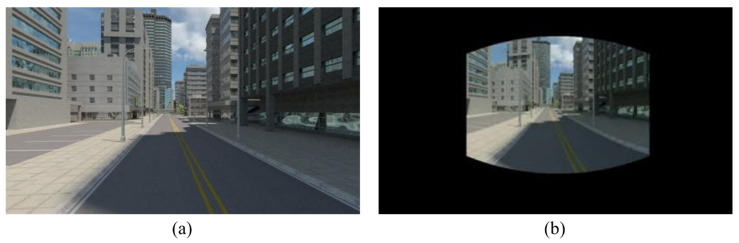
(**a**) The perspective source image from “Multi-FOV” synthetic datasets. (**b**) The projection result of perspective source image from “Multi-FOV” synthetic datasets obtained using the proposed method.

In order to obtain the credible test results of the proposed 360° SLAM system, we used perspective image sequences of the “Multi-FOV” synthetic datasets [16] and performed the equirectangular projection for the input image type of the proposed system. Figure 29b depicts the projection result (1886 × 942) from the perspective image (640 × 480) in Figure 29a provided by the dataset.

**Figure 30:**
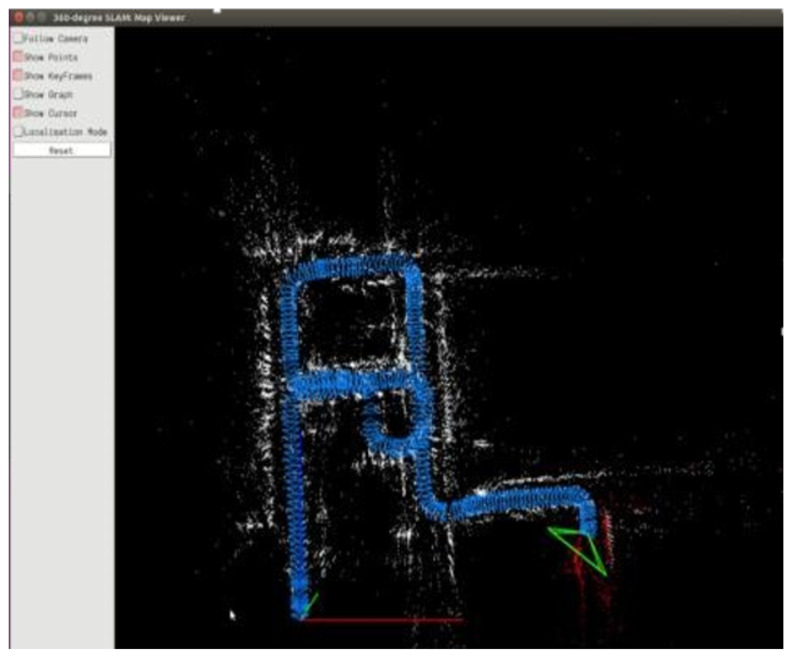
The result of map built from the dataset.

The equirectangular panorama was input to the proposed system, and the trajectory was saved for comparing with the ground truth. Figure 30 presents the result of the map built from the “Multi-FOV” synthetic datasets.

A total of 2500 images were used as the sequential inputs to the proposed system, and there was no loop detected. In other words, the final map never performed loop closure. Although the loop can be observed in the final map shown in Figure 30, the camera practically moved in opposite direction on the same road. With the availability of front view only, the system could not recognize the road passing from the opposite direction as the scene the camera captured was completely different.

**Figure 31:**
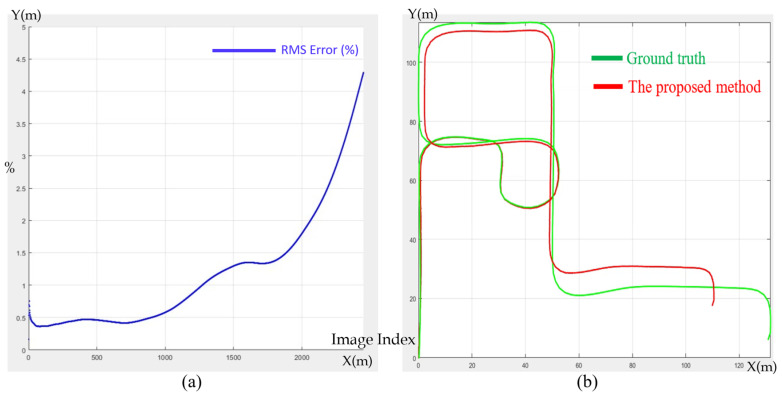
(**a**) Accumulated RMS in percentage with corresponding image index. (**b**) The trajectory comparison with the ground truth.

Figure 31a illustrates the accumulated root-mean-square (RMS) error as a percentage with the corresponding image index, and Figure 31b shows that the pose error increased when the camera made a quarter turn. The accumulated error was less than 1% when the camera moved 250 m, and increased when the image index passed through ~1200 m (at the left top corner of the map). Overall, the accumulated error was 4.25% after the camera finished moving 500 m without doing loop closure. The sudden spike in the RMS error shown in Figure 31a was due to the cumulated error of the proposed SLAM method applied on the Multi-FOV synthetic dataset [16] for 360° V-SLAM. Since the path in the synthetic dataset was not a closed loop, we could not perform loop closure to adjust the trajectory of the path to minimize the path error, which caused an increase in the RMS error at the end of the trajectory. Since the proposed method can be adapted to the cases of 360° FOV video maps and SLAM, there is no limitation on the pose constraints, as autonomous vehicles are designed to search and localize themselves in the map.

### 4.3. Experimental Results

**Figure 32:**
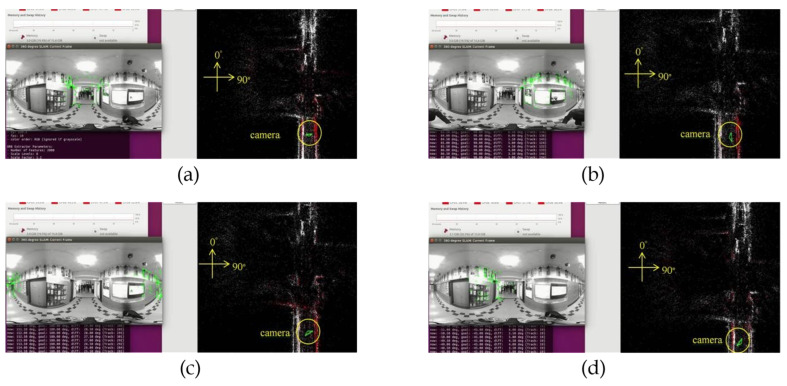
Localization results: (**a**) 0° yaw; (**b**) 88° yaw; (**c**) 155° yaw; (**d**) 315° yaw.

Figure 32a–d demonstrate the localization result of the proposed method at different yaw degrees covering all the FOVs. The corresponding camera pose is shown in the map viewer on the right side of respective figures.

**Figure 33:**
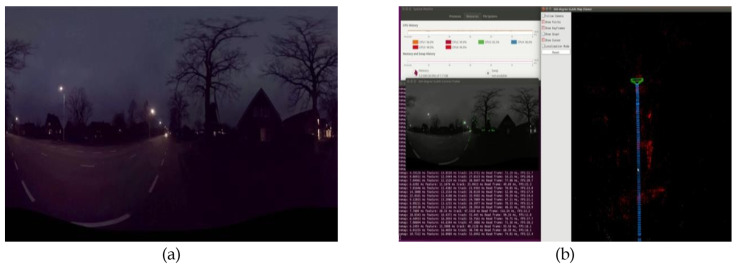
(**a**) Night-scene of raw equirectangular panorama. (**b**) Result of the proposed 360° SLAM system for night-scene.

For indoor and outdoor daytime scenes, the proposed method worked efficiently. In the night scene shown in Figure 33a, initialization of the SLAM system was more difficult than for the daytime due to the low luminance. However, once the initialization was finished, the proposed 360° SLAM system tracked the features well, even with the low brightness, as the proposed SLAM system is a feature-based SLAM method, and the FOV is wide enough to estimate the camera pose with more details in the scene. The results of the proposed method are presented in Figure 33b.

Additionally, some of the in-house demo videos showing the implementation of the proposed work can be found at https://www.youtube.com/watch?v=E2g6mFzIoos (accessed on 3 September 2022), https://studio.youtube.com/video/C3vxSVNXhhI/edit (accessed on 24 May 2023), and https://studio.youtube.com/video/M0GNTTQz09Y/edit (accessed on 24 May 2022).

### 4.4. Comparison

**Figure 34:**
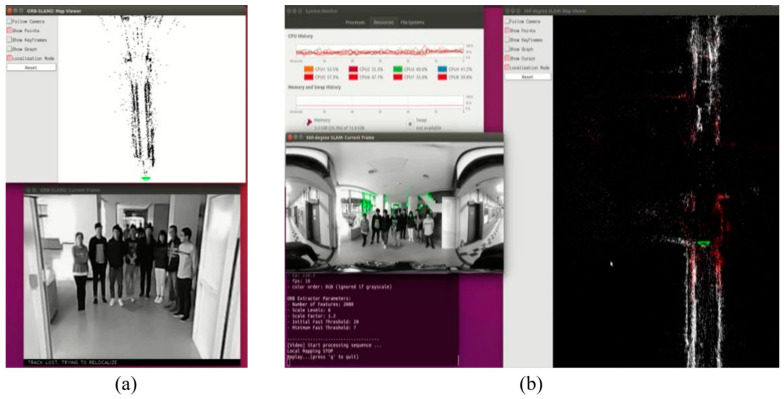
(**a**) Monocular method SLAM gets lost when people stand in front of the camera. (**b**) The 360° SLAM can successfully localize at 0° yaw shown in green-colored localized points.

For autonomous driving vehicle applications, the camera may be occluded by obstacles, people, or other objects. Figure 34a shows the results of the monocular SLAM method, demonstrating that, when the image with only 94° FOV was occupied with people standing in front of the camera, the monocular SLAM method was lost. On the other hand, Figure 34b illustrates that, even with many people standing in front of the camera, the proposed 360° SLAM system could still estimate the camera pose by using features located as highlighted in green feature points at other regions in the image due to the 360° FOV. A comparison of the two results revealed that the proposed 360° SLAM system is more robust than the existing methods since the panorama can completely cover the maximum FOV.

## 5. Conclusions

A general-purpose, robust, fast, and real-time simultaneous localization and mapping (SLAM) system was proposed in this paper. The challenges faced by the camera-based methods are variations in weather, lighting conditions, and the environment, along with finding stable and unique features to estimate the camera pose. The proposed method uses an equirectangular panorama as input image to achieve the 360° FOV. In addition, the camera calibration, fast unwrapped algorithm, stitching, and blending are implemented to achieve an efficient and effective real-time performance. The perspective transformation with any yaw degree given is adopted to decrease the area of feature extraction in order to save time. At the same time, the ORB feature extraction is sped up fivefold with the GPU acceleration. The functions such as 360° binary map saving, loading, and online updating make the proposed method more convenient, flexible, and stable to use in real-time applications. The proposed method achieves not only better performance and a wider FOV, but also better accuracy. The precision of open-loop localization is a challenge for the proposed system. Our proposed method was implemented both on a desktop machine and on an nVidia Jetson TX2 with GPU, achieving 20 fps for 1416 × 708 panorama video input on the embedded systems. The test result of the proposed method was more robust than the monocular SLAM method, and the open-loop accuracy was 1% accumulated RMS error for 250 m and 4.25% for 500 m.

To overcome certain limitations of the proposed method in the process of localization in the proposed SLAM algorithm, perspective transformation is performed only on a specific region of panorama. However, the other regions that may also possess meaningful information are missed, thereby not considering more details which can be used for the camera pose estimation. Therefore, in the future work, a new bundle-adjustment algorithm for a fisheye camera model may be helpful to tackle this challenge. Moreover, when combined with other depth sensors, such as LiDAR or radar, the accuracy can be improved, and absolute distance information can be provided in the real world.

## Figures and Tables

**Table 1:** Four operation modes in the proposed SLAM system.

	Saving the Map	Not Saving the Map
**Map existing**	Normal mode (with online updating)	Localization mode
**Map not found**	Map establishment mode	SLAM mode

**Table 2:** Global parameters.

**Number of cameras**	2
**Circular fisheye FOV**	200° (each camera)
**Camera input resolution**	1024 × 768 (each camera)
**Equirectangular panorama resolution**	1416 × 708
**Blending width**	40 pixels
**Perspective transformation parameters**	720 × 480 with 130° FOV
**Orb features extracted each frame**	2000

**Table 3:** Specifications and performance of the proposed algorithm on the PC without GPU.

Processor	Intel i7-3770 @ 3.40 GHZ
**GPU**	None
**Memory**	16GB RAM
**Video In**	File input
**Operation System**	Ubuntu 14.04
**Performance**	20 fps

**Table 4:** Specifications and performance of the proposed algorithm on the nVIDIA with GPU.

**Processor**	ARM Cortex-A57 (quad-core) @ 2 GHz + NVIDIA Denver2 (dual-core) @ 2 GHz
**GPU**	256 core Pascal @ 1300 MHz
**Memory**	8 GB 128 bit LPDDR4 @ 1866 MHz
**Video In**	File input
**OS**	Ubuntu 16.04.5 LTS
**Performance**	20 fps

## Data Availability

Not applicable.
